# Drug-Related Problems Among Peritoneal Dialysis Patients: A 12-Year Retrospective Cohort Study

**DOI:** 10.7759/cureus.69700

**Published:** 2024-09-19

**Authors:** Hui Yin Tay, Farida Islahudin, Yi Yun Siaw, Wu Ching Wong, Nor Asyikin Mohd Tahir, Shahnaz Shah Firdaus Khan

**Affiliations:** 1 Department of Pharmacy, Hospital Tengku Ampuan Rahimah, Klang, MYS; 2 Center for Quality Management of Medicine, Faculty of Pharmacy, Universiti Kebangsaan Malaysia, Kuala Lumpur, MYS; 3 Department of Nephrology, Hospital Tengku Ampuan Rahimah, Klang, MYS

**Keywords:** clinical pharmacy services, continuous ambulatory peritoneal dialysis, drug-related problems (drps), medication therapy adherence clinic, peritoneal dialysis (pd), pharmacists-led clinic, dialysis

## Abstract

Introduction

Studies regarding drug-related problems (DRPs) can be found in other diseases, but data are lacking among peritoneal dialysis (PD) populations. Despite advancements in PD care, there remains a significant gap in understanding and addressing DRPs in the PD population. DRPs can lead to serious consequences, including medication errors, adverse reactions, and nonadherence, affecting patient outcomes and healthcare costs.

Aim

The aim of this study was to identify the prevalence of DRPs, types, causes, interventions performed, acceptance of interventions, and outcomes of DRPs among patients undergoing PD. In addition to this, the study sought to identify factors associated with DRPs in the PD population.

Methods

This single-center retrospective study recruited adult PD patients with at least one medication from January 2009 until November 2021. Pharmacy medication therapy adherence clinic (MTAC) clinical activity sheets were reviewed, and DRPs were classified based on the Pharmaceutical Care Network Europe Classification (PCNE) v9.1. The PCNE system consists of five essential domains: Problems (P), Causes (C), Interventions (I), Acceptance of the Intervention (A), and Outcomes (O). As part of the pharmacists’ MTAC activities, DRPs were meticulously documented. Three pharmacists initially gathered and examined these recorded DRPs. Each identified DRP was then classified according to the type of problem, the underlying cause, any intervention performed to address the DRP, the level of acceptance, and the resulting outcome. Subsequently, these classifications were reviewed by two independent pharmacists to ensure accuracy and consistency.

Results

Out of 562 patients, 70.6% (n = 397) were on more than 10 drugs. Most patients (n = 520, 92.5%) had at least one DRP. From the 3,333 DRPs identified, the most common were effects of drug treatment not optimal (n = 1,595, 47.8%), followed by untreated symptoms (n = 843, 25.3%) and adverse drug events (n = 730, 21.9%). The main cause of the suboptimal treatment effect was patients’ noncompliance (n = 891, 55.9%). For untreated symptoms, the main cause was no drug prescribed despite existing indications (n = 789, 93.6%). Interventions for DRPs were at either prescriber level (n = 2,064, 61.9%), patient-level (n = 1,244, 37.3%), or at other levels, such as with nurses (n = 25, 0.8%). Prescribers accepted 83% (n = 1713) of interventions suggested by pharmacists. Overall, 73.2% (n = 2,439) of DRPs were resolved. Number of medications (b = 0.223, 0.102-0.345) and number of MTAC visits (b = 0.381, 0.344-0.419) were predictive factors of the number of DRPs (p < 0.001).

Conclusion

There is a high prevalence of DRPs in PD patients. Pharmacists play an important role in detecting, intervening, and resolving DRPs to improve patients’ outcomes.

## Introduction

The pharmacist-managed medication therapy adherence clinic (MTAC) was an initiative started in Malaysia in 2004 by the Pharmaceutical Services Division, Ministry of Health, as part of the clinical pharmacy services [1,2]. Due to the tremendous increase of dialysis patients over the past 10 years, peritoneal dialysis (PD) has also been slowly gaining popularity and acceptance [3], which has led to the implementation of a peritoneal dialysis-medication therapy adherence clinic (PD-MTAC). The aim of the PD-MTAC is to optimize medication therapy and clinical outcome. This is performed through various roles and responsibilities during the PD-MTAC, in which pharmacists routinely assess medication adherence, ensure fulfillment of partial filling of medications, counsel patients on medication, identify drug-related problems (DRPs), and address the need for DRP interventions with close discussion of attending nephrologists, as well as monitor clinical outcome of medications.

DRPs are prevalent among end-stage kidney disease (ESKD) patients, especially those on dialysis [4]. They are known to interfere with desired health outcomes [5], potentially complicating outcomes for PD patients, increasing patient mortality and morbidity, and escalating healthcare costs [6]. Previous studies have shown that DRP increases the risk of hospital admissions [7]. Among 395 ambulatory hemodialysis (HD) patients, pharmacists identified a total of 1,593 DRPs, averaging one DRP for every 6.5 medications reviewed, highlighting the high risk of DRPs in this patient group [8]. In a study of stage 5 chronic kidney disease (CKD) geriatric patients, 93.2% had at least one DRP, with pharmacists identifying 394 DRPs in 103 patients, averaging 3.8 ± 2.4 DRPs per patient [9]. A six-month prospective study found that 78.6% of CKD patients admitted to hospitals experienced DRPs, averaging 1.93 ± 0.873 DRPs per patient [10].

Among the common types of DRPs among CKD patients were inappropriate laboratory investigations, drug therapy without apparent indication, and dosing errors [8]. Other studies found that needing extra drug therapy, nonadherence to medications, doses too low [10], medications with unclear indications, inappropriate drug administrations, and noncompliance to guidelines [9] were the most prevalent DRPs among CKD patients. DRPs in CKD, particularly those on PD, can have severe consequences. These include worsening kidney function, increased hospitalization risk, diminished quality of life due to side effects, and even increased mortality. By identifying and addressing DRPs, this study aims to improve patient outcomes and reduce the overall burden of CKD.

Previous research has highlighted the prevalence of DRPs among CKD patients, particularly those undergoing HD. However, there is a notable gap in understanding DRPs among PD patients, despite the growing number of individuals on this treatment modality. This study aims to address this gap by comprehensively investigating the types, causes, and outcomes of DRPs in the PD population. By identifying factors associated with DRPs and evaluating the impact of pharmacist interventions, this study seeks to contribute to the optimization of care for this high-risk patient group. Ultimately, the findings will inform strategies to prevent and manage DRPs, thereby improving the overall health and quality of life for PD patients. Therefore, the objective of this study was to identify the prevalence of DRPs, types, causes, interventions performed, acceptance of interventions, and outcomes of DRPs among patients undergoing PD. In addition to this, the study sought to identify factors associated with DRPs in the PD population.

## Materials and methods

Study design

This retrospective study focused on PD patients attending the MTAC at Hospital Tengku Ampuan Rahimah, a prominent 1,094-bed tertiary institution in Malaysia. Over the past decade, the number of patients with ESKD on PD has steadily risen. By 2021, Malaysia reported 5,897 individuals undergoing PD, based on the Malaysian National Renal Registry 2021 [3], with 252 active PD patients receiving care at Hospital Tengku Ampuan Rahimah during the same year. The target population for this study included all PD patients who were seen by pharmacists in the PD-MTAC from 2009 to 2021. Patients included in the study were adults ≥18 years old with at least one prescribed medication. Patients with missing data were excluded, as complete data was required to fully assess DRP classifications. Archived files were also excluded, as these files were not accessible to the researchers. However, the number of these was small compared to the overall sample size.

Sample size

The sample size was calculated using the Raosoft® Calculator [11]. The calculated sample size was at least 361 patients with a 5% margin of error and a 95% CI. The sample size calculation was based on a population of PD patients in Malaysia of 5,897 in 2021 [3] and an assumed response distribution of 50%.

Data collection

All data were collected from patients’ MTAC files and categorized into two sections. The first section included demographic data such as gender, ethnicity, age, number of comorbidities, number of medications, and number of MTAC visits. The second section comprised MTAC data on DRPs classified according to the Pharmaceutical Care Network Europe (PCNE) Classification v9.1. The PCNE Classification categorizes each DRP into Problems (P), Causes (C), Interventions (I), Acceptance of the interventions (A), and Outcomes (O).

Altogether, there are three primary domains for the problem (P1, P2, and P3). P1 refers to Treatment effectiveness, P2 relates to Treatment safety, and P3 Others. These are further divided into six subdomains (P1.1, P1.2, P1.3, P2.1, P3.1, and P3.2). For causes (C), there are nine primary domains (C1-C9) and 43 subdomains of DRPs. This includes C1 Drug selection, C2 Drug form, C3 Dose selection, C4 Treatment duration, C5 Dispensing, C6 Drug use process, C7 Patient related, C8 Patient transfer related, and C9 Others. Interventions (I) were categorized as I0 No intervention, I1 At the prescriber level, I2 At the patient level, I3 At the drug level, or I4 Others, and further divided into 17 subdomains. Data regarding the acceptance of interventions by pharmacists were subcategorized as A1 Intervention accepted, A2 Intervention not accepted, or A3 Others if acceptance status was unknown. The status of each identified DRP was categorized as O0 Problem status unknown, O1 Problem solved, O2 Problem partially solved, or O3 Problem not solved. DRPs identified at the final MTAC visit of the study were categorized as O0 Problem status unknown, as no follow-up was available by the end of the study period.

As part of MTAC, pharmacists are trained to identify DRPs and record all steps involved, including the reason for the DRP and plans related to overcoming DRPs, as well as monitoring and recording the outcomes during follow-up sessions. Therefore, for the study, a total of three pharmacists were involved in the data collection of recorded DRPs. They gathered data from MTAC activity sheets and were involved in classifying each DRP based on PCNE classifications: Problems (P), Causes (C), Interventions (I), Acceptance of the interventions (A), and Outcomes (O) as well as their sub-domains. If there were discrepancies in the DRP classifications, the pharmacists clarified and discussed these issues among themselves. Training and briefings were provided to ensure that all team members had a clear and mutual understanding of DRP classifications. The collected data were then reviewed by two independent pharmacists. This study focused exclusively on actual DRPs identified by pharmacists during PD-MTAC visits, excluding potential DRPs from its scope.

Data analysis

All data were analyzed using IBM SPSS Statistics for Windows, Version 26.0 (Released 2019; IBM Corp., Armonk, NY, USA). Normally distributed data were expressed as mean ± SD, while non-normally distributed data were expressed as median and IQR. PCNE classifications of DRPs, problems, causes, interventions carried out, acceptance, and outcomes of the interventions, were analyzed descriptively as frequency and percentages. Factors associated with the number of DRPs were analyzed using simple linear regressions. Gender, ethnicity, age, number of comorbidities, number of medications, and number of MTAC visits were categorized as independent variables, whereas the number of DRPs was the dependent variable. In the single linear regression, variables that had a p-value of <0.25 were further analyzed in the multiple linear regression. Multicollinearity was checked and not found. A p-value of <0.05 from the multiple linear regression was considered significant.

## Results

Demographic data and clinical characteristics

A total of 562 patients were recruited. The majority of patients were male (n = 327, 58.2%) and of Malay ethnicity (n = 413, 73.5%). The average age of the patients was 52.2 (±14.3) years, with an average of 2.6 (±1.2) comorbidities. Patients visiting the MTAC took an average of 10.9 ± 2.6 medications, with a median of 8 (IQR 4-14.3) visits throughout the study period (Table 1).

**Table 1:** Demographic data and clinical characteristics (n = 562) DM, diabetes mellitus; DRPs, drug-related problems; HPT, hypertension; IHD, ischemic heart disease

Parameters (n = 562)	Value
Age, mean (±SD), in years	52.2 ± 14.3
<60 years old	367 (65.3)
60 years old	195 (34.7)
Gender, n (%)	
Male	327 (58.2)
Female	235 (41.8)
Ethnicity, n (%)	
Malay	413 (73.5)
Chinese	74 (13.2)
Indian	69 (12.3)
Others	6 (1.1)
Number of comorbidities, mean (SD)	2.6 ± 1.2
Comorbidities, n (%)	
DM	358 (63.7)
HPT	480 (85.4)
Dyslipidemia	182 (32.4)
IHD	84 (14.9)
Anemia	212 (37.7)
Number of medications, mean (±SD)	10.9 ± 2.6
Number of visits, median (IQR)	8 (4-14.25)
Number of DRPs, median (IQR)	5 (2-9)

Types and causes of DRPs identified

A total of 3,333 DRPs were identified among PD-MTAC patients, with the majority (n = 520, 92.5%) experiencing at least 1 DRP. Among these, the most common (n = 1,595, 47.8%) was P1.2: Effect of drug treatment not optimal, followed by P1.3: Untreated symptoms or indication (n = 843, 25.3%) and P2.1: Adverse drug event (possibly) occurring (n = 730, 21.9%) (Table 2).

**Table 2:** Types of DRPs among PD-MTAC patients (n = 3,333) DRPs, drug-related problems; PD-MTAC, peritoneal dialysis-medication therapy adherence clinic

Problems		Frequency, n	Percentage, %
P1	Treatment effectiveness (total)	2,441	73.2
P1.1	No effect of drug treatment despite correct use	3	0.1
P1.2	Effect of drug treatment not optimal	1,595	47.8
P1.3	Untreated symptoms or indication	843	25.3
P2	Treatment safety (total)	730	21.9
P2.1	Adverse drug event (possibly) occurring	730	21.9
P3	Other	162	4.9
P3.1	Unnecessary drug treatment	140	4.2
P3.2	Unclear problem/complaints	22	0.7
Total		3,333	100

The main cause of DRPs was due to C7 Patient-related factors (1,265, 380%), followed by C3 Dose selection (893, 26.8%) and C1 Drug selection (1,086, 32.6%) (Table 3).

**Table 3:** Causes of DRPs among PD-MTAC patients (n = 3,333) DRPs, drug-related problems; PD-MTAC, peritoneal dialysis-medication therapy adherence clinic; TDM, therapeutic drug monitoring

Causes		Frequency, n	Percentage, %
C1	Drug selection (total)	1,086	32.6
C1.1	Inappropriate drug according to guidelines/formulary	63	1.9
C1.2	No indication of drug	151	4.5
C1.3	Inappropriate combination of drugs, or drugs and herbal	19	0.6
C1.4	Inappropriate duplications	12	0.4
C1.5	No or incomplete drug treatment in spite of existing indication	818	24.5
C1.6	Too many different drugs/active ingredients prescribed for indication	23	0.7
C2	Drug form (total)	40	1.2
C2.1	Inappropriate drug form/formulation (for this patient)	40	1.2
C3	Dose selection (total)	893	26.8
C3.1	Drug dose too low	295	8.9
C3.2	Drug dose of a single active ingredient too high	395	11.9
C3.3	The dosage regimen not frequent enough	57	1.7
C3.4	Dosage regimen too frequent	138	4.1
C3.5	Are timing instructions wrong, unclear, or missing	8	0.2
C4	Treatment duration (total)	7	0.2
C4.1	Duration of treatment too short	2	0
C4.2	Duration of treatment too long	5	0.2
C5	Dispensing (total)	2	0
C5.1	Prescribed drugs not available	2	0
C6	Drug use process (total)	18	0.5
C6.1	Inappropriate timing of administration or dosing intervals by a health professional	4	0.1
C6.2	Drug under-administered by a health professional	6	0.2
C6.3	Drug over-administered by a health professional	2	0
C6.4	Drug not administered at all by a health professional	6	0.2
C7	Patient related (total)	1265	38
C7.1	Patient intentionally uses/takes less drug than prescribed or does not take the drug at all for whatever reason	929	27.9
C7.2	Patient uses/takes more drug than prescribed	60	1.8
C7.4	Patient decides to use unnecessary drug	6	0.2
C7.5	Patient takes food that interacts	1	0
C7.6	Patient stores drug inappropriately	4	0.1
C7.7	Inappropriate timing or dosing intervals	83	2.5
C7.8	Patients unintentionally administers/uses the drug in a wrong way	142	4.3
C7.9	Patient physically unable to use drug/form as directed	36	1.1
C7.10	Patient unable to understand instructions properly	4	0.1
C9	Other (total)	22	0.6
C9.1	No or inappropriate outcome monitoring (including TDM)	4	0.1
C9.2	Other causes; specify	16	0.5
C9.3	No obvious cause	2	0
Total		3,333	100

**Figure 1:**
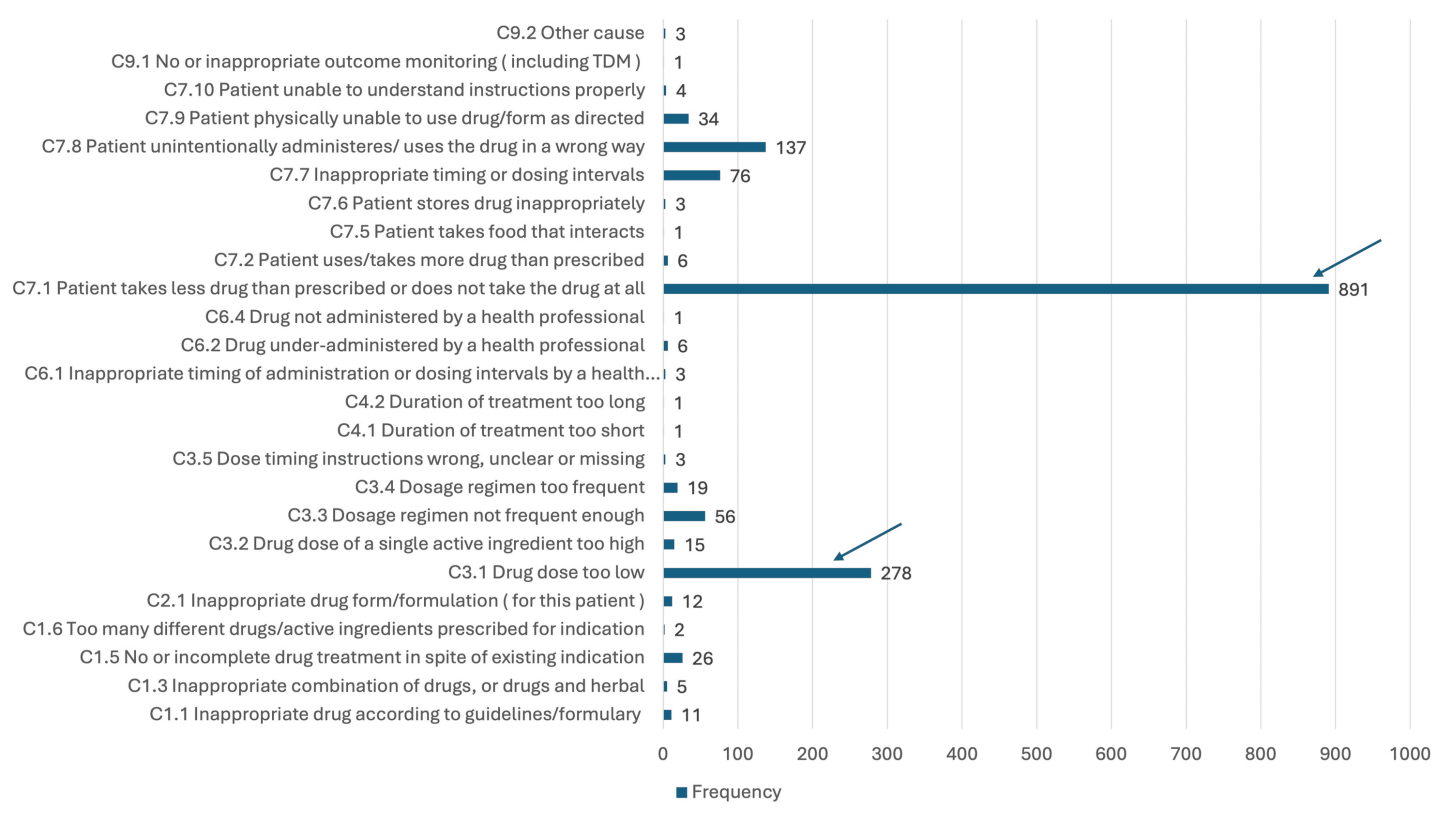
Causes for P1.2 effect of drug treatment not optimal X = frequency; Y = causes

A sub-analysis into the causes of the main problems P1.2, P1.3, and P2.1 showed that for P1.2: Effect of drug treatment not optimal (n = 1,595, 47.8%), was primarily caused by C7.1: Patient intentionally uses or takes less drug than prescribed or does not take the drug at all (n = 891, 55.9%). This was mainly due to nonadherence to calcium carbonate, antihypertensives, and subcutaneous erythropoietin injection. The second most common cause for suboptimal drug effect was C3.1: Drug dose prescribed by prescribers being too low (n = 278, 17.5%) (Figure 1).

**Figure 2:**
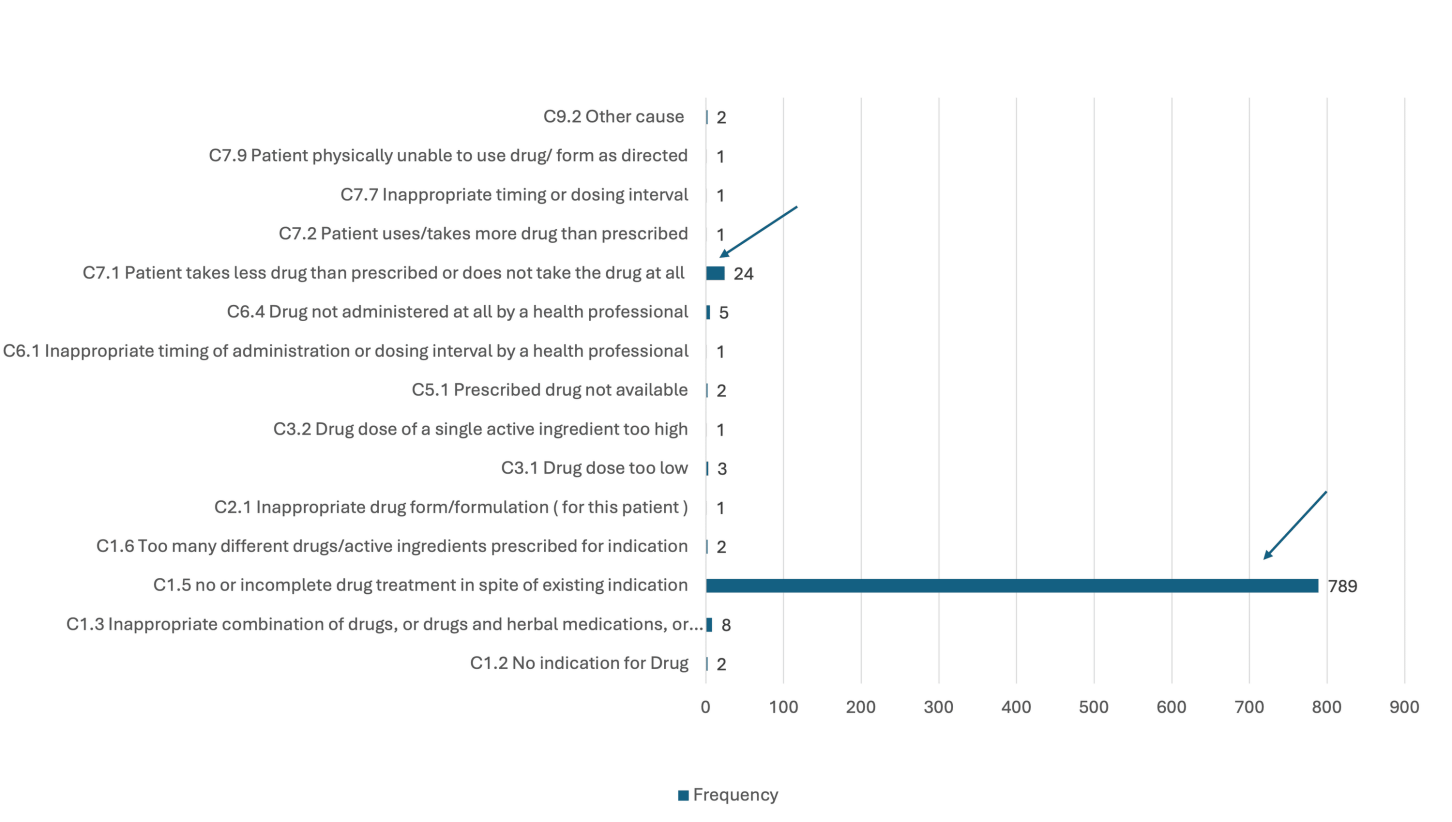
Causes for DRP P1.3 untreated symptoms or indication X = frequency; Y = causes DRP, drug-related problem

P1.3: Untreated symptoms or indications (n = 843, 25.3%) were most often caused by C1.5: No or incomplete drug treatment despite existing indication (n = 789, 93.6%), followed by C7.1 Patient takes less drug than prescribed or does not take the drug at all (24, 2.8%) (Figure 2).

**Figure 3:**
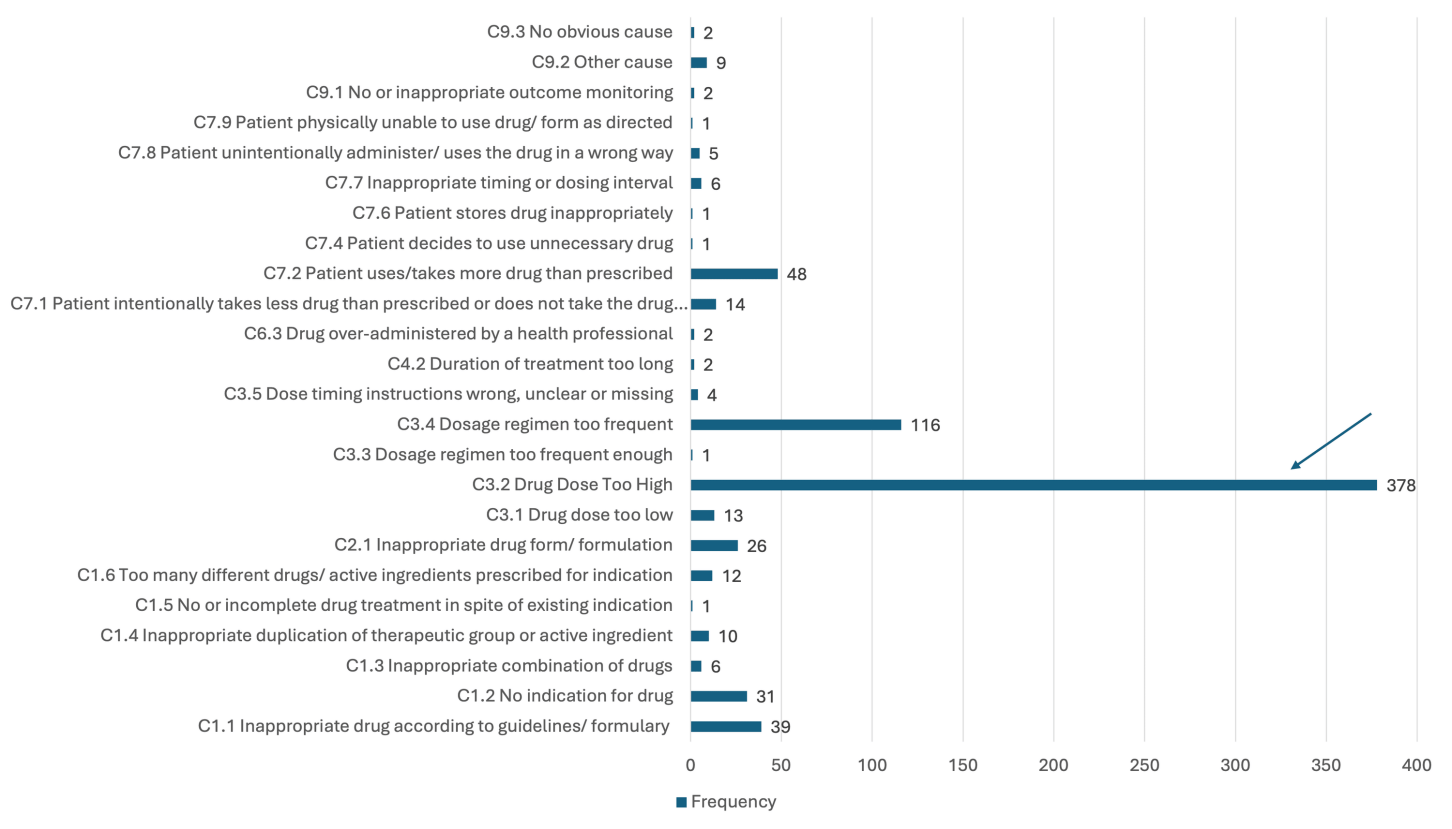
Causes for DRP P2.1 ADR (possibly) occurring X = frequency; Y = causes ADR, adverse drug reaction; DRP, drug-related problem

P2.1: Adverse drug event (possibly) occurring was the third most common DRP (n = 730, 21.9%). Half of the causes of P2.1 were C3.2: Drug dose too high (n = 378, 51.7%), followed by C3.4: Drug regimen too frequent (n = 116, 15.9%) (Figure 3).

Interventions and acceptance of interventions by pharmacists 

Out of the 3,333 identified DRPs, most interventions were conducted at the prescriber level (I1, n = 2,064, 61.9%), involving requests to change the patient’s drug regimen by the pharmacist (Table 4).

**Table 4:** Types of intervention of DRPs among PD-MTAC patients (n = 3,333) DRPs, drug-related problems; PD-MTAC, peritoneal dialysis-medication therapy adherence clinic

Intervention		Frequency, n	Percentage, %
I1	Intervention at prescriber level (total)	2,064	61.9
I1.4	Intervention proposed to prescriber	2,064	61.9
I2	Intervention at patient level (total)	1,244	37.3
I2.1	Patient (drug) counseling	1,244	37.3
I3^a^	Intervention at drug level (total)	1,713	100
I3.1	Drug changed to …	67	3.9
I3.2	Dosage changed to …	744	43.4
I3.3	Formulation changed to …	5	0.3
I3.4	Instructions for use changed to …	12	0.7
I3.5	Drug paused or stopped	181	10.6
I3.6	Drug started	704	41.1
I4	Other Intervention or activity (total)	25	0.8
Total		3,333	100

Of the 2,064 interventions proposed to prescribers, 1713 (83%) were accepted. Accepted interventions by prescribers were further categorized under I3: At drug level to specify types of interventions conducted at the drug level. The most common interventions accepted by prescribers (I1) included I3.2: Dosage changed (n = 744, 43.3%), I3.6: Starting a new drug (n = 704, 41.1%), I3.5: Drug paused or stopped (n = 181, 10.6%), I3.1: Drug changed (n = 67, 3.9%), I3.4: Instructions for use changed (n = 12, 0.7%), and I3.2: Formulation changed (n = 5, 0.3%) (Table 4).

A total of 1,244 interventions (37.3%) were carried out at the patient level (I2), involving medication counseling. Other interventions (I4, n = 25, 0.8%) included interventions with nurses regarding patients’ drug regimens (Table 4).

Among the 3,333 DRPs and interventions conducted by pharmacists, a significant number (n = 2,961, 88.8%) were accepted by either prescribers or patients (A1.1-A1.4) (Table 5). Only 9.4% (n = 315) of interventions were not accepted (A2: Intervention not accepted), while 1.7% (n = 57) had no information regarding acceptance (A3: Others).

**Table 5:** Acceptance of intervention of DRPs among PD-MTAC patients (n = 3,333) DRPs, drug-related problems; PD-MTAC, peritoneal dialysis-medication therapy adherence clinic

Acceptance of intervention		Frequency, n	Percentage, %
A1	Intervention accepted (total)	2,961	88.8
A1.1	Intervention accepted and fully implemented	2,667	80
A1.2	Intervention accepted, partially implemented	200	6
A1.3	Intervention accepted but not implemented	82	2.5
A1.4	Intervention accepted, implementation unknown	12	0.3
A2	Intervention not accepted (total)	315	9.5
A2.2	Intervention not accepted, no agreement	315	9.5
A3	Other (total)	57	1.7
A3.1	Intervention proposed, acceptance unknown	57	1.7
Total		3,333	100

Outcome of the interventions

The majority (n = 2,439, 73.2%) of DRPs were successfully resolved (O1.1). However, a total of 626 (18.8%) DRPs remained unsolved (O3.1-O3.3), primarily due to O3.1: Lack of cooperation from patients (n = 348, 10.4%) where patients remained noncompliant, or O3.2: Lack of cooperation from prescribers (n = 261, 7.8%). There were 218 (6.5%) DRPs with an unknown status (O0.1) (Table 6).

**Table 6:** Outcomes of DRPs among PD-MTAC patients (n = 3,333) DRPs, drug-related problems; PD-MTAC, peritoneal dialysis-medication therapy adherence clinic

Outcome		Frequency, n	Percentage, %
O0.1	Problem status unknown	218	6.5
O1.1	Problem totally solved	2,439	73.2
O2.1	Problem partially solved	29	0.8
O3.1	Problem not solved, lack of cooperation of patient	348	10.4
O3.2	Problem not solved, lack of cooperation of prescriber	261	7.8
O3.3	Problem not solved; intervention not effective	17	0.5
O3.4	No need or possibility to solve problem	21	0.6
Total		3,333	100

Factors associated with DRPs

Results from the simple and multiple linear regression showed factors associated with number of DRPs in PD patients were number of medications (b = 0.228, 95% CI 0.107-0.348, p < 0.001) and number of MTAC visits (b = 0.381, 95% CI 0.344-0.419, p < 0.001) (Table 7).

**Table 7:** Factors affecting number of DRPs in PD-MTAC patients ^b^ Crude regression coefficient DRPs, drug-related problems; MTAC, medication therapy adherence clinic; PD-MTAC, peritoneal dialysis-medication therapy adherence clinic

Factors	b (95% CI)^b^	p-value
Simple linear regression (reference)		
Female (male)	-0.283 (-1.130, 0.564)	0.512
Age (years)	-0.026 (-0.055, 0.003)	0.077
Ethnicity (Indian)		
Malay	0.304 (-0.988, 1.596)	0.644
Chinese	1.054 (-0.608, 2.717)	0.213
Number of comorbidities	0.213 (-0.135,0.562)	0.23
Number of medications	0.333 (0.176, 0.490)	<0.001
Number of MTAC visits	0.388 (0.350, 0.425)	<0.001
Multiple linear regression		
Number of MTAC visits	0.381 (0.344, 0.419)	<0.001
Number of medications	0.223 (0.102, 0.345)	<0.001

For every one increment in number of medications, DRPs among PD patients increased by 22.8%. Besides that, number of MTAC visits was also a predictive factor for number of DRPs, where an increment of 1 MTAC visit increased the number of DRPs by 38%. The predicted number of DRPs is equal to 0.382 (number of MTAC visits) + 0.228 (number of medications) -0.433.

## Discussion

PD patients usually have multiple comorbidities that lead to the use of a high number of medications [12]. Evidently, the current work showed a high pill burden among PD patients, averaging 11 medications similar to other CKD studies [6,9]. The high medication burden often leads to a higher risk of DRP, with more than 90% of the study population presenting with at least one DRP, similar to previous work among CKD patients that ranged between 78% and 97% [6,9,10,13]. This high rate of DRP causes a concern among PD patients, as it could lead to poorer clinical outcomes.

The most common DRP among our PD patients was the suboptimal effect of medication. This was mainly caused by intentionally using or taking less drug than prescribed or patients not taking the drug at all. Adherence is a common problem among chronically ill patients with multiple medications [14]. Among CKD patients, the lack of adherence was often reported for medications such as calcium carbonate, erythropoietin injections, and insulin [15-17]. Among Malaysian CKD patients, similar results were reported, with up to 48% of patients finding it hard to tolerate phosphate binders due to their unpleasant taste and difficulty in injecting insulin [16]. The difficulty in taking medications should be addressed, as this could potentially affect the clinical outcomes of PD patients. Another cause for the suboptimal effect of medication was drug doses that were too low, often reported with the use of vitamin D analogs such as calcitriol and alfacalcidol. Patients with normal calcium and phosphate levels but increased intact parathyroid hormone needed to have their vitamin D analog dose increased [18]. However, this was not done for several PD patients, and pharmacists were noted to discuss the issue with prescribers, suggesting an increase in the dose of vitamin D.

Untreated symptoms or indications was also frequently reported as a DRP among the current PD patients, similar to previous work [10,13]. More than 90% of the untreated symptoms or indications were due to the lack of drug treatment in spite of existing indications. Medication omission errors were found to be common among patients with a high pill burden [19], especially at centers that use manual prescriptions such as our study site [20]. One of the main concerns is the need to transfer medications from case notes to the required prescriptions, and the risk of missing out on medications is high due to the high number of medications taken by each patient. However, during the MTAC sessions, pharmacists were able to identify these problems and approached prescribers whenever omission errors were noted. Furthermore, upon reviewing patient laboratory results and medication lists, if there were any indications that necessitated a drug, pharmacists were noted to discuss this with prescribers, and suggestions for additions of drugs were performed.

During the study duration, PD-MTAC pharmacists were noted to actively intervene by suggesting dose changes, addition of new drugs, or stopping an unnecessary drug. It was noted that prescribers accepted 83% of the pharmacists’ interventions, which was an exceptionally high rate [21]. One of the common reasons for the nonacceptance of pharmacists’ interventions in the current work was because the prescribers prefer to repeat a laboratory test before implementing the change suggested. As such, more than three-quarters of the DRPs identified were resolved successfully, comparable to previous work [22]. However, unresolved DRPs were mainly due to continuous nonadherence despite pharmacists’ counseling, often observed among dialysis patients [14,23]. This suggests that further interventions are required to improve adherence in some patients. It was, however, noted that a number of patients did not give sufficient cooperation, leading to the need for behavioral counseling. The lack of motivation to overcome the general dislike of taking medicine was likely to remain among some patients [23]. Emphasis should be placed on enhancing motivation during medication counseling of PD patients using techniques such as motivational interviewing that have been shown to improve patients’ adherence to medications [24-26].

Due to the high number of DRPs among PD patients, identifying factors of DRPs is vital. It was noted that the number of medications and number of MTAC visits were significant predictors of DRPs. The need to review patients with a high pill burden has been similarly reported [10,13,27,28], due to the risk of higher errors. Evidently, in the current work, there were 140 unnecessary drugs, either due to drugs being prescribed without indications, too many drugs prescribed for the same indications, inappropriate duplication of therapeutic group, or duration of treatment that was too long. Among CKD patients, unnecessary drug treatment has similarly been demonstrated [9], highlighting the need for frequent review. This further emphasizes that with frequent MTAC visits and drug reviews, which are also predictors of DRP among PD patients, optimum drug management is ensured. PD-MTAC pharmacists were able to identify various DRPs during MTAC visits due to the ability to reconcile all medications taken by PD patients from various clinics, which were not accessible by prescribers. The continuous review of PD patients was also essential in identifying adherence among patients, which tends to reduce over time [15,29-30].

To that end, the current work demonstrates the need for clinical pharmacy services among PD patients. The strength of the study lies in the large number of PD patients who received PD-MTAC care that spanned a 12-year period. The large sample size of PD patients in our study enhances the robustness of our findings by ensuring that the results are representative and generalizable to the broader PD population. By including a substantial number of patients over a 12-year period, we were able to capture a diverse range of DRPs and the variability in pharmacist interventions across different patient demographics and clinical settings. This extensive data set provides a comprehensive overview of DRPs, which strengthens the reliability of the identified trends and patterns. Moreover, the long study duration allows for a thorough examination of the long-term impact of clinical pharmacy services. The ability to follow up on identified DRPs over this extended period enables us to assess not only the immediate effectiveness of pharmacist interventions but also their sustained outcomes and acceptance by prescribers. The combination of a large sample size and a lengthy study period significantly enhances the validity of our findings by providing a more accurate and comprehensive assessment of the impact of clinical pharmacy services on DRPs in the PD population.

There were, however, a few limitations of the study, of which the first was being a single-centered study. Future work could be performed in various other PD-MTAC centers to ensure a comprehensive review of DRPs. Due to the retrospective nature of the work, the data collected was dependent on the availability and accuracy of patients’ data in PD-MTAC files. DRPs that were not identified and reported may not be collected, leading to a possible lack of data. Some data may have been lost, as a few patients had missing data, and files that were archived were not included in the study, introducing a potential for bias. For example, among the biases is that the excluded files may contain concentrated data in a particular group, which may lead to some confounding bias and inaccuracy in overall findings. However, it is noteworthy that the volume of missing and archived data was minimal relative to the overall sample size. Thus, while generalizability should be approached with care, the study still provides valuable insights into the efficacy of clinical pharmacy services in the context of PD.

## Conclusions

DRPs are highly prevalent among PD patients due to the high number of medications taken by this group of patients. PD-MTAC visits are therefore necessary after each clinic visit to minimize DRPs through close monitoring of patients. This ensures optimal medication management and promotes a more positive clinical outcome in the long run. The study’s longitudinal design, coupled with a large sample size, provides robust evidence supporting the efficacy of PD-MTAC visits. The longitudinal approach allowed a unique insight into the persistence and resolution of DRPs over time, while the large sample size enhanced the generalizability of our findings. This design reinforces the conclusion that regular, comprehensive medication reviews are essential for minimizing DRPs and improving patient outcomes. Specifically, 88.8% of the interventions by pharmacists were accepted by either prescribers or patients; and 73.2% of DRPs resolved following PD-MTAC visits. Pharmacists, with their specialized expertise in medication therapy management, are uniquely positioned to address these issues effectively. Their involvement in identifying and resolving DRPs not only minimizes adverse effects but also optimizes drug therapy and enhances overall patient health. By proactively identifying and resolving DRPs, pharmacists can contribute significantly to enhancing the quality of life and overall health of PD patients.
